# Strategies to attenuate micro-vascular obstruction during P-PCI: the randomized reperfusion facilitated by local adjunctive therapy in ST-elevation myocardial infarction trial

**DOI:** 10.1093/eurheartj/ehw136

**Published:** 2016-05-04

**Authors:** Sheraz A. Nazir, Gerry P. McCann, John P. Greenwood, Vijay Kunadian, Jamal N. Khan, Islam Z. Mahmoud, Daniel J. Blackman, Martin Been, Keith R. Abrams, Lorraine Shipley, Robert Wilcox, A.A. Jennifer Adgey, Anthony H. Gershlick

**Affiliations:** 1 Department of Cardiovascular Sciences, University of Leicester and NIHR Leicester Cardiovascular Biomedical Research Unit, Glenfield Hospital, Groby Road, Leicester LE3 9QP, UK; 2 Multidisciplinary Cardiovascular Research Centre, Leeds Institute of Cardiovascular and Metabolic Medicine, University of Leeds, Leeds, UK; 3 Institute of Cellular Medicine, Newcastle University and Cardiothoracic Centre, Freeman Hospital, Newcastle upon Tyne Hospitals NHS Foundation Trust, Newcastle upon Tyne, UK; 4 Department of Cardiovascular Imaging, Division of Imaging Sciences & Biomedical Engineering, Rayne Institute, BHF Excellence Centre, St Thomas' Hospital, King's College London, London, UK; 5 Department of Cardiology, University Hospitals Coventry and Warwickshire NHS Trust, Coventry, UK; 6 Department of Health Sciences, School of Medicine, University of Leicester, Leicester, UK; 7 Faculty of Medicine & Health Sciences, Queen's Medical Centre, Nottingham, UK; 8 Heart Centre, Royal Victoria Hospital, Belfast, UK

**Keywords:** Acute myocardial infarction, Microvascular obstruction, Ischaemia-reperfusion injury, Magnetic resonance Imaging, Adenosine, Nitroprusside

## Abstract

**Background:**

Microvascular obstruction (MVO) following primary percutaneous coronary intervention (PPCI) treatment of ST-segment elevation myocardial infarction (STEMI) contributes to infarct expansion, left ventricular (LV) remodelling, and worse clinical outcomes. The REFLO-STEMI trial tested whether intra-coronary (IC) high-dose adenosine or sodium nitroprusside (SNP) reduce infarct size and/or MVO determined by cardiac magnetic resonance (CMR).

**Methods and results:**

REFLO-STEMI, a prospective, open-label, multi-centre trial with blinded endpoints, randomized (1:1:1) 247 STEMI patients with single vessel disease presenting within 6 h of symptom onset to IC adenosine (2–3 mg total) or SNP (500 μg total) immediately following thrombectomy and again following stenting, or to standard PPCI. The primary endpoint was infarct size % LV mass (%LVM) on CMR undertaken 24–96 h after PPCI (*n* = 197). Clinical follow-up was to 6 months. There was no significant difference in infarct size (%LVM, median, interquartile range, IQR) between adenosine (10.1, 4.7–16.2), SNP (10.0, 4.2–15.8), and control (8.3, 1.9–14.0), *P* = 0.062 and *P* = 0.160, respectively, vs. control. MVO (% LVM, median, IQR) was similar across groups (1.0, 0.0–3.7, *P* = 0.205 and 0.6, 0.0–2.4, *P* = 0.244 for adenosine and SNP, respectively, vs. control 0.3, 0.0–2.8). On per-protocol analysis, infarct size (%LV mass, 12.0 vs. 8.3, *P* = 0.031), major adverse cardiac events (hazard ratio, HR, 5.39 [1.18–24.60], *P* = 0.04) at 30 days and 6 months (HR 6.53 [1.46–29.2], *P* = 0.01) were increased and ejection fraction reduced (42.5 ± 7.2% vs. 45.7 ± 8.0%, *P* = 0.027) in adenosine-treated patients compared with control.

**Conclusions:**

High-dose IC adenosine and SNP during PPCI did not reduce infarct size or MVO measured by CMR. Furthermore, adenosine may adversely affect mid-term clinical outcome.

**Clinical Trial registration:**

ClinicalTrials.gov Identifier: NCT01747174; https://clinicaltrials.gov/ct2/show/NCT01747174

**See page 1920 for the editorial comment on this article (doi:10.1093/eurheartj/ehw186)**

## Introduction

Primary percutaneous coronary intervention (PPCI) is the default reperfusion therapy for ST-segment elevation myocardial infarction (STEMI).^1^ However, residual mortality (up to 12% at 6 months^1^) and morbidity may be partially attributable to sub-optimal microvascular perfusion.^2,3^ Microvascular obstruction (MVO) can occur in up to 70% patients when detected with cardiovascular magnetic resonance (CMR) imaging.^4,5^ Microvascular obstruction may contribute to infarct expansion, adverse left ventricular (LV) remodelling and adverse clinical outcomes, independent of infarct size.^4,5^

Various strategies have been proposed to attenuate MVO^6^ and have included calcium channel blockers, nicorandil, atrial natriuretic peptide, glycoprotein IIb/IIIa (GPIIb/IIIa) inhibitors, thrombolytics, and, perhaps most studied, vaso-active agents such as sodium nitroprusside (SNP)^2,7,8^ and adenosine.^8–12^ Adenosine inhibits neutrophil-related processes central to the evolution of MVO and in a canine model, reduced ischaemia-reperfusion injury (IRI), limited infarct size, and improved ventricular function.^13^ Sodium nitroprusside is a direct nitric oxide donor that mediates potent arteriolar vasodilation, inhibits platelet adhesion and promotes anti-inflammatory processes,^14^ which effectively reduce MVO in animal IRI models.^15^ Previous clinical trials with these agents varied in their design and they mostly lacked a sensitive method to detect MVO, which may have contributed to the multiple conflicting results seen.^7–9,12,16,17^ Uncertainty remains regarding the potential therapeutic impact of these agents.^1^

We hypothesized that, in patients undergoing PPCI for STEMI, appropriate doses of intra-coronary (IC) adenosine or SNP delivered distally to the coronary bed, and again after stent deployment, was the optimal administration that would reduce infarct size and MVO as determined by the sensitive measure of in-patient CMR.

## Methods

### Study design

The trial design and methods have been published previously.^16^ Briefly, REFLO-STEMI was a multi-centre, prospective, randomized, open-label, controlled trial, with blinded endpoint analysis, conducted in accordance with the Helsinki Declaration. Ethical approval for the study (reference 11/H0405/10) was obtained from the National Research Ethics Service (UK). ST-segment elevation myocardial infarction patients with single vessel disease and thrombolysis in myocardial infarction (TIMI) flow grades 0–1 in the IRA were enrolled between October 2011 and April 2014 in four regional cardiac centres in the UK. All patients >18 years age, presenting within 6 h of symptom onset, with ST-segment elevation ≥2 mm in ≥2 contiguous leads and with a baseline corrected QT interval (QTc) <450 ms on admission electrocardiogram (ECG) were eligible and *assented*.^16^ Full eligibility criteria are shown in Supplementary material online, *Table S1*.

### Randomization and treatment

Patients were randomized using a 24-h interactive voice recognition service with concealed allocation. All patients were pre-treated with aspirin 300 mg and prasugrel 60 mg or ticagrelor 180 mg^1^ as well as bivalirudin 0.75 mg/kg bolus plus infusion of 1.75 mg/kg/h until completion of PPCI or continued for 4 h if clinically indicated. Bivalirudin, recommended in all patients, was standard of care in the enrolling centres at the time of trial initiation. Allocation was 1:1:1 to adenosine, SNP, or control (standard PPCI alone) with stratification by ‘symptoms to balloon either less than or ≥3 h’ and ‘anterior infarction or not’. Manual thrombectomy was mandated in all patients and the thrombectomy catheter then used to deliver the first drug bolus (adenosine 1 mg or SNP 250 μg) IC distal in the coronary bed, after thorough flushing of the catheter. Immediately following stent deployment, and providing a repeat QTc was <450 ms (or <60 ms increased over baseline value), the second dose (adenosine 1 mg if IRA was the right coronary artery otherwise 2 mg or SNP 250 μg) was delivered over 1 min via the guide catheter (see *Figure 2*).

### Study outcomes

**Figure 1:**
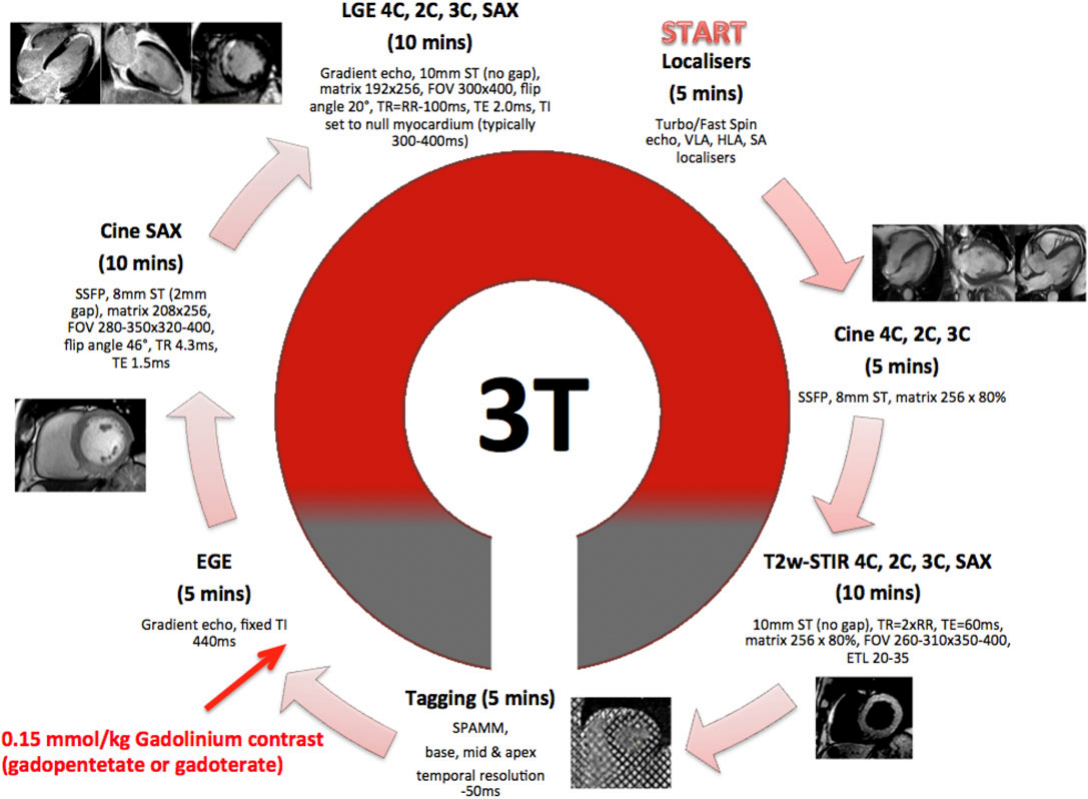
Cardiac magnetic resonance Protocol. 4C, 2C, 3C = 4,2,3-chamber long-axis views; SPAMM, spatial magnetization modulation; ETL, echo train length; FOV, field of view; LV, left ventricle; SAX, short axis; SSFP, steady-state-free precession; ST, slice thickness; TI, inversion time; TE, echo time; TR, repetition time.

Cardiac magnetic resonance was performed at 24–96 h on a 3.0T scanner (*Figure 1*) to determine infarct size (primary endpoint).^16^ Secondary outcomes included: MVO extent assessed on early- and late-gadolinium enhanced (EGE, LGE) images and the presence of intra-myocardial haemorrhage (IMH) on CMR; ST-segment resolution (STR) on ECG performed 90 min post-PPCI; major adverse cardiovascular events (death, MI, heart failure, target lesion revascularization, and stroke; major adverse cardiac events, MACE) within 6 months. Important adverse events and study outcome measures are defined online (see Supplementary material online, *Tables S5* and *S6*, respectively). A clinical events committee, blinded to patient details and treatment allocation, reviewed and adjudicated key trial adverse events using original source documents. An independent DSMB, with an independent statistician, periodically reviewed trial conduct and outcome data.

### Cardiac magnetic resonance analysis

Cardiac magnetic resonance was performed as previously described^16^ and analysis, blinded to all patient details and treatment allocation, was undertaken offline in a central core lab (University of Leicester) using *cmr42* (Circle Cardiovascular Imaging, Calgary, Canada). Early-gadolinium enhanced and LGE images were acquired after 1–3 and 10–15 min, respectively, following 0.15 mmol/kg gadolinium-DTPA (*Magnevist*, Bayer, Germany) administration using a single-shot or segmented inversion-recovery gradient-echo sequence. Early- and late-MVO (E-MVO, L-MVO) were defined as hypoenhancement within the infarct territory on EGE and LGE imaging, respectively. Left ventricular volumes, infarct size, myocardial oedema (area at risk, AAR), intra-myocardial haemorrhage (IMH), and MSI were determined as described previously.^16^

### Sample size

To detect a reduction in infarct size from 20 to 15% of LV mass, assuming a standard deviation of 10%,^18–22^*α* of 0.05 and two tailed, with 80% power, a total of 192 patients were required. Allowing for a drop-out rate of 20% between PPCI and CMR resulted in a final sample size requirement of 240 patients.

### Statistical methods

Primary analysis was by intention-to-treat with a secondary per-protocol analysis in those patients who received both doses of the investigational drugs. Continuous variables, including infarct size, were investigated for normality and were log-transformed when found to be non-normally distributed. Normally distributed continuous variables were expressed as mean ± standard deviation and compared using *t*-tests and analysis of variance (ANOVA). Non-normally distributed data were presented as median (25th–75th quartiles) and compared using non-parametric methods (Mann–Whitney or Kruskal–Wallis). Comparison between groups for categorical outcomes was undertaken using χ^2^ tests. Potential significant confounders of infarct size (age, sex, diabetes, anterior MI, ischaemia time and collateral blood flow to the infarct territory determined by the *Rentrop* score^23^) were assessed using a forward selection procedure (statistical significance level of 5%). Multivariable analysis using linear regression was used to adjust for significant confounders. Time-to-event Cox proportional hazards regression models were used to assess predictors of first MACE (both at 30 days and at the study end) and to obtain unadjusted and adjusted hazard ratios (HRs) with 95% confidence intervals (CIs) for adenosine/SNP vs. control. In addition, actuarial event rates of MACE by 1 and 6 months for each intervention were reported with 95% CIs. The assumption of proportional hazards was assessed by a global hypothesis test.^24^ No formal adjustment for multiple hypothesis testing was used, but *P*-values were interpreted cautiously. All statistical analyses were performed using SPSS (Version 20.0. Armonk, NY: IBM Corporation) and R (Version 3.1.2. The R Foundation for Statistical Computing, Vienna).

## Results

**Figure 2:**
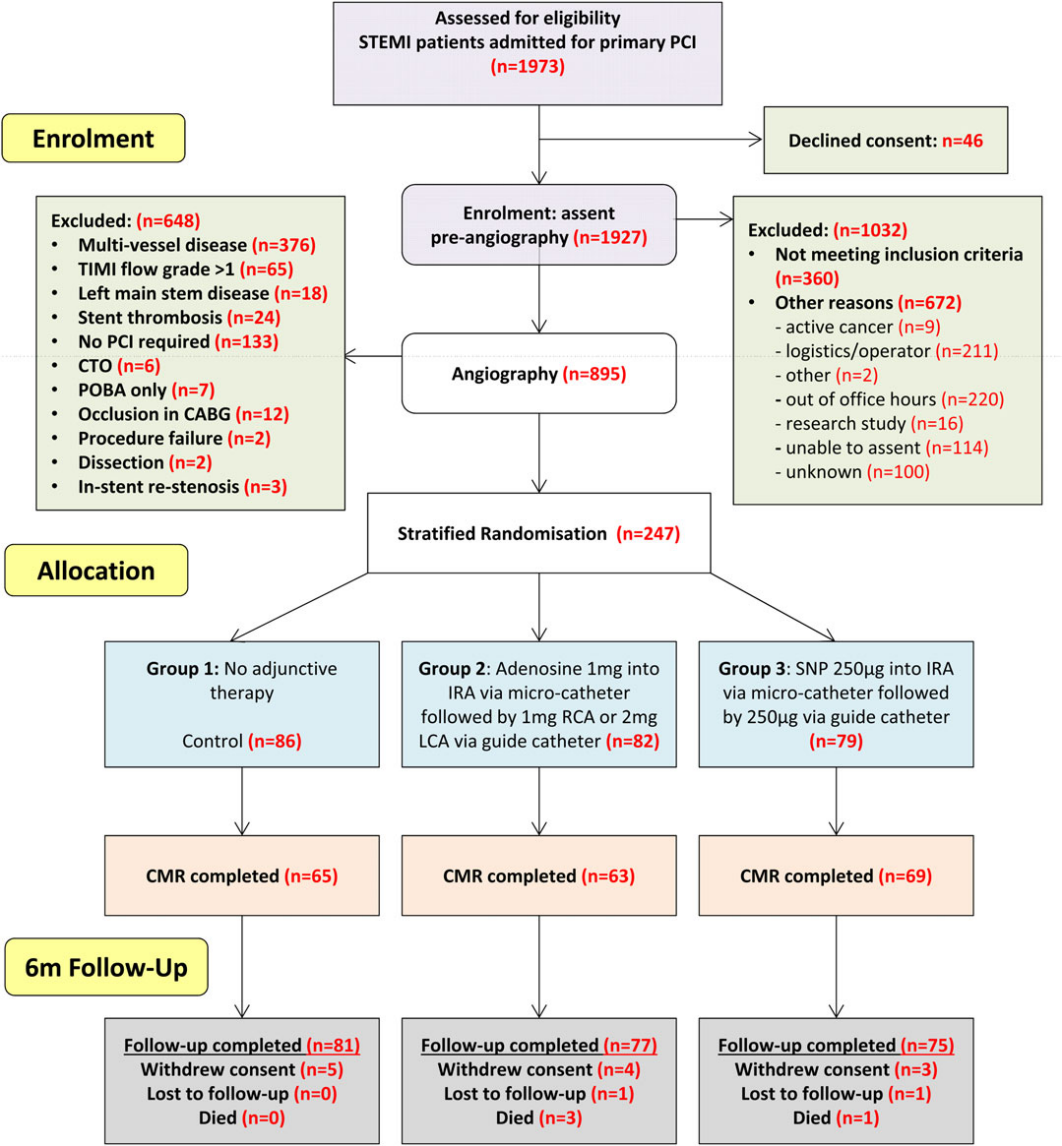
Study recruitment flowchart (CONSORT). CABG, coronary artery bypass graft; CTO, chronic total occlusion; IRA, infarct-related artery; LCA, left coronary artery; POBA, plain old balloon angioplasty; PPCI, primary percutaneous coronary intervention; RCA, right coronary artery; SNP, sodium nitroprusside; TIMI, thombolysis in myocardial infarction.

Patient recruitment is outlined in *Figure 2*. Two hundred and forty-seven patients were randomized with 207 (84%) undergoing CMR. Ten patients did not complete the CMR (due to claustrophobia or musculoskeletal discomfort) so that the primary outcome of infarct size was assessed in 197 patients (80% of those randomized).

### Baseline characteristics

Baseline characteristics for randomized patients are presented in *Table 1* (see Supplementary material online, *Table S7* for patients completing CMR by treatment allocation). There were no significant differences in characteristics of those randomized and those who completed CMR.

**Table 1:** Demography of the total trial population

Characteristics	Adenosine (*n* = 82)	SNP (*n* = 79)	Control (*n* = 86)	*P*-value
Clinical				
Age (years)	57.9 ± 12.8	60.5 ± 13.0	59.5 ± 11.2	0.406
Male	65/82 (79.3)	66/79 (83.5)	64/86 (74.4)	0.355
Hypertension	23/82 (28.0)	26/79 (32.9)	22/86 (25.6)	0.574
Current smoking	47/82 (57.3)	41/79 (51.9)	45/86 (52.3)	0.332
Diabetes	6/82 (7.3)	12/79 (15.2)	9/86 (10.5)	0.274
Hypercholesterolaemia	17/82 (20.7)	23/79 (29.1)	11/86 (12.8)	0.035
Previous MI	0/81 (0)	3/79 (3.8)	3/86 (3.5)	0.219
Killip class >1	3/82 (3.7)	4/79 (5.1)	4/86 (4.7)	0.905
Total ischaemia time (min)	159 (124–221)	150 (122–201)	145 (105–196)	0.169
BMI (kg/m^2^)	27.5 (25.0–30.1)	26.1 (24.3–30.8)	27.3 (24.4–30.6)	0.659
SBP (mmHg)	137.5 ± 25.1	133.0 ± 23.4	135.1 ± 23.3	0.505
DBP (mmHg)	86.6 ± 19.2	81.8 ± 16.4	80.5 ± 17.0	0.067
HR (beats/min)	74.0 ± 17.3	71.9 ± 14.8	71.2 ± 14.6	0.487
Cr clearance (mL/min/1.73 m^2^)	98.1 ± 28.1	93.4 ± 29.1	92.7 ± 25.6	0.401
Anti-platelet use				
Aspirin	82/82 (100.0)	79/79 (100.0)	86/86 (100.0)	1.000
Clopidogrel	16/82 (19.5)	13/79 (16.5)	12/86 (14.0)	0.625
Prasugrel	38/82 (46.3)	33/79 (41.8)	40/86 (46.5)	0.790
Ticagrelor	28/82 (34.1)	33/79 (41.8)	34/86 (39.5)	0.591
Medication on discharge				
β-Blocker	72/78 (92.3)	69/75 (92.0)	73/81 (90.1)	0.867
ACE-inhibitor/A2RB	74/78 (94.9)	75/75 (100.0)	79/81 (97.5)	0.133
Statin	77/78 (98.7)	74/75 (98.7)	80/81 (98.8)	0.999
Infarct-related artery				
LAD—proximal	19/82 (23.2)	18/79 (22.8)	20/86 (23.3)	0.997
LAD—other	13/82 (15.9)	15/79 (19.0)	14/86 (16.3)	0.848
LCX	10/82 (12.2)	13/79 (16.5)	18/86 (20.9)	0.314
RCA	40/82 (48.8)	33/79 (41.8)	34/86 (39.5)	0.455
TIMI flow pre-PPCI				
0–1	80/81 (98.8)	72/79 (91.1)	83/86 (96.5)	0.057
2	1/81 (1.2)	5/79 (6.3)	1/86 (1.2)	0.078
3	0/81 (0.0)	2/79 (2.5)	2/86 (2.3)	0.367
Thrombus score				
4–5	75/81 (92.6)	72/79 (91.1)	81/86 (94.2)	0.754

Values are mean ± SD or *n* (%).

ACE-inhibitors/A2RB, angiotensin-converting enzyme inhibitors/angiotensin II receptor blockers; BMI, body mass index; CABG, coronary artery bypass graft; CAD, coronary artery disease; Cr, creatinine; DBP, diastolic blood pressure; HR, heart rate; LAD, left anterior descending coronary artery; LCX, left circumflex coronary artery; PCI, percutaneous coronary intervention; RCA, right coronary artery; SBP, systolic blood pressure; TA, thrombus aspiration; TIMI, thrombolysis in myocardial infarction.

### Angiography and primary percutaneous coronary intervention procedure details

Radial access, thrombectomy, and drug-eluting stent use were uniformly high (*Table 2*). Intra-procedural complications were similar across all groups. However, the incidence of transient atrio-ventricular (AV) block not requiring pacing was greater in the control arm. There was a low incidence of AV block requiring pacing with the study drugs (adenosine 2.4% vs. SNP 1.3% vs. 0% in control arm). The rate of transient hypotension (not requiring vasopressor or intra-aortic balloon-pump) was almost three-fold greater (16.5 vs. 5.8%, *P* = 0.028) in the SNP group compared with control.

**Table 2:** Procedural (angiographic, electrocardiographic, and enzymatic) data and intra-procedural complications according to treatment group

Characteristics	Adenosine (*n* = 82)	SNP (*n* = 79)	Control (*n* = 86)	*P*-value^a^	*P*-value^b^
Procedural data					
Radial approach	70 (85.4)	70 (88.6)	79 (91.9)	0.184	0.481
Thrombectomy	81 (98.8)	75 (98.7)	80 (93.0)	0.118	0.122
DES implantation	73 (89.0)	72 (91.1)	81 (94.2)	0.226	0.452
Number of stents	1.0 (1.0–2.0)	1.0 (1.0–2.0)	1.0 (1.0–2.0)	0.613	0.790
Diameter of stented segment (mm)	3.5 (3.0–3.5)	3.5 (3.0–3.5)	3.0 (3.0–3.5)	0.465	0.649
Length of stented segment (mm)	26.0 (18.0–39.0)	23.0 (18.0–38.0)	24.0 (18.0–34.5)	0.585	0.833
Electrocardiographic					
Baseline maximal sum of ST-segment elevation	9.0 (6.0–12.0)	9.0 (5.0–13.0)	9.0 (5.0–13.0)	0.931	0.686
Post-PCI maximal sum of ST-segment elevation	2.0 (0.0–5.0)	2.0 (0.0–5.0)	2.0 (0.0–4.0)	0.684	0.472
STR >70%	56 (68.3)	48 (60.8)	56 (65.1)	0.662	0.562
Enzymatic					
Peak CK (mg/dL)	1559 (601–2804)	1171 (430–2259)	1336 (511–2632)	0.601	0.393
Intra-procedural complications					
Transient AV block not requiring pacing	7 (8.5)	2 (2.5)	10 (11.6)	0.507	0.034
AV block requiring pacing	2 (2.4)	1 (1.3)	0 (0.0)	0.237	0.479
Transient hypotension not requiring vasopressor drugs or IABP	5 (6.1)	13 (16.5)	5 (5.8)	0.938	0.028
Hypotension requiring vasopressor drugs or IABP	5 (6.1)	3 (3.8)	6 (7.0)	0.818	0.499
Ventricular tachycardia/fibrillation	5 (6.1)	3 (3.8)	5 (5.8)	0.938	0.722

Values are mean ± SD, median (interquartile range), or *n* (%).

AV, atrio-ventricular; CK, creatine kinase; DES, drug-eluting stent; IABP, intra-aortic balloon-pump; STR, ST-segment resolution; TMPG, tissue myocardial perfusion grade.

^a^Adenosine vs. Control.

^b^SNP vs. Control.

### Drug delivery

Both doses of investigational drugs were administered in 66/82 (80%) and 53/79 (67%) patients (adenosine and SNP, respectively). The commonest reasons for withholding the second dose were QTc prolongation in 30 (adenosine: 9; SNP: 21) and hypotension in 3 (adenosine: 2; SNP: 1) patients. Failure to cross the lesion or deploy a stent and prohibiting intra-procedural complications accounted for the remainder.

### Angiographic, electrocardiogram, and enzymatic assessment of myocardial injury

There was no difference in the occurrence of post-PPCI TIMI flow grade <3 between groups (*Table 2*). Likewise ECG (STR>70%) assessment of microvascular tissue perfusion was similar across the groups. There was no statistically significant difference between groups in peak creatine kinase levels.

### Cardiac magnetic resonance assessment of myocardial injury: primary end point

There was no statistically significant difference in the primary outcome measure of unadjusted infarct size between groups (*Table 3*). On multivariable regression analysis, adjusting for significant confounders, mean infarct size was increased in the adenosine group (mean difference 2.73, 95% CI: −0.18 to 5.64, *P* = 0.066) when compared with controls. This was not seen in the SNP group.

**Table 3:** Cardiac magnetic resonance data according to treatment group

Characteristic	Adenosine, *n* = 63	SNP, *n* = 69	Control, *n* = 65	*P*-value^a^	*P*-value^b^	*P*-value^c^
Time from MI to CMR (h)	49.0 (28.4–75.0)	49.7 (26.2–76.1)	49.0 (38.0–74.8)	0.773	0.843	0.881
Primary endpoint^d^						
Infarct size (%LVM)	10.1 (4.7–16.2)	10.0 (4.2–15.8)	8.3 (1.9–14.0)	0.062	0.160	0.133
Microvascular injury						
Presence of E-MVO (*n*, %)	41/60 (68.3)	42/59 (71.2)	38/63 (60.3)	0.452	0.254	0.416
E-MVO (%LVM)	1.2 (0.0–5.2), *n* = 60/82	1.0 (0.0–5.0), *n* = 59/79	1.4 (0.0–4.3), *n* = 63/86	0.637	0.770	0.891
Presence of L-MVO (*n*, %)	43/63 (68.3)	52/69 (75.4)	37/65 (56.9)	0.205	0.029	0.074
L-MVO (%LVM)	1.0 (0.0–3.7)	0.6 (0.0–2.4)	0.3 (0.0–2.8)	0.205	0.244	0.368
Presence of IMH (*n*, %)	20/38 (52.6)	19/43 (44.2)	16/38 (42.1)	0.358	0.850	0.619
	*n* = 34	*n* = 38	*n* = 37			
Salvage						
AAR (%LVM)	30.6 ± 12.2	34.7 ± 14.4	30.3 ± 11.5	0.907	0.152	0.266
MSI (%)	60.2 ± 23.3	63.6 ± 24.7	67.5 ± 23.3	0.188	0.477	0.429
	*n* = 63	*n* = 71	*n* = 68			
Function and volumes						
LVEDVI (mL/m^2^)	91.3 ± 16.1	87.0 ± 16.9	84.4 ± 14.6	0.011	0.336	0.044
LVESVI (mL/m^2^)	52.3 ± 13.8	49.1 ± 12.7	46.1 ± 11.6	0.006	0.155	0.023
LVMI (g/m^2^)	59.1 ± 11.5	56.0 ± 11.2	53.6 ± 9.2	0.003	0.174	0.014
EF (%)	43.2 ± 7.9	43.9 ± 6.5	45.7 ± 8.0	0.080	0.165	0.155

Values are mean ± SD or median (interquartile range) unless otherwise stated.

AAR, area at risk; BSA, body surface area; EF, ejection fraction; IMH, intra-myocardial haemorrhage; LV, left ventricular; LVEDVI, LV end-diastolic volume indexed to BSA; LVESVI, LV end-systolic volume indexed to BSA; LVM, LV mass; LVMI, LVM indexed to BSA; MI, myocardial infarction; MSI, myocardial salvage index; E- or L-MVO, early- or late-microvascular obstruction.

^a^Adenosine vs. Control.

^b^SNP vs. Control.

^c^All groups.

^d^Primary endpoint—comparison via independent *t*-test on log-transformed scale.

Late-MVO was present in 67% patients and was significantly more prevalent in the SNP arm vs. control (75.4 vs. 56.9%, *P* = 0.029). However, the extent of L-MVO was small and not significantly different between any groups. Other CMR parameters of microvascular injury (E-MVO and IMH) were also similar between groups and none of the potential confounders were identified as being of statistical importance by the forward selection procedure. Diagnostic quality T2-weighted (oedema) imaging was only obtainable in 109 patients (55%). There were no significant differences in AAR or MSI between groups.

Left ventricular end-diastolic and end-systolic volumes indexed to BSA (LVEDVI, LVESVI) were significantly increased with adenosine compared with control and this was accompanied by a non-significant reduction in ejection fraction (EF). Left ventricular volumes and function were similar in the SNP and standard PPCI arms (*Table 3*).

### Clinical outcomes

**Figure 3:**
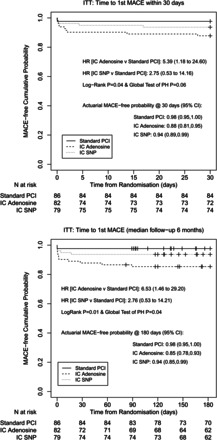
Kaplan–Meier graphs showing clinical outcome. Clinical outcomes at 30 days (top) and 6 months (bottom)*.* HR, hazard ratio; ITT, intention-to-treat; MACE, major adverse cardiac events; PH, proportional hazards.

Two hundred and thirty-three (94%) patients completed follow-up (12 patients withdrew consent/refused follow-up and 2 patients were lost to follow-up). There was a significant increase in MACE at 6 months (15.6 vs. 2.5%, *P* = 0.004) in the adenosine-treated group compared with control (see *Figure 3*) driven by incidence of heart failure (10.4 vs. 1.2%, *P* = 0.016); HR 6.53 (95% CIs 1.46–29.2), *P* = 0.01. When adjusted for potential confounders, the observed MACE difference remained with similar hazard ratios with *P*-values: *P* = 0.018 at 30 days and *P* = 0.01 at 6 months (see Supplementary material online, *Table S9*). There was no statistically significant difference in bleeding between groups (see *Table 4*).

**Table 4:** Clinical events (first event) to 6 months according to treatment group

Characteristics	Adenosine (all subjects) (*n* = 82)	SNP (all subjects) (*n* = 79)	Control (all subjects) (*n* = 86)	*P*-value^a^	*P*-value^b^
MACE	12 (15.0, 7.0–22.0)^c^	5 (6.0, 1.0–15.0)^c^	2 (2.0, 0.0–5.0)^c^	0.004	0.261
Death	1 (1.2)	1 (1.3)	0 (0.0)	0.488	0.479
TIA/stroke	1 (1.2)	1 (1.3)	0 (0.0)	0.488	0.479
MI	2 (2.4)	1 (1.3)	1 (1.2)	0.614	1.000
HF	8 (9.8)	2 (2.5)	1 (1.2)	0.016	0.607
TLR	0 (0.0)	0 (0.0)	0 (0.0)	1.000	1.000
Composite of death, MI, and HF	11 (13.4)	4 (5.1)	2 (2.3)	0.009	0.428
No. of patients with >1 event	3 (3.7)	1 (1.3)	1 (1.2)	0.359	1.000
Bleeding					
All bleeding	4 (4.9)	2 (2.5)	5 (5.8)	1.000	0.446
Fatal bleeding	0 (0.0)	0 (0.0)	0 (0.0)	1.000	1.000

HF, heart failure; MACE, major adverse cardiac events; MI, myocardial infarction; TIA, transient ischaemic attack; TIMI, thrombolysis in MI; TLR, target lesion revascularization.

^a^Adenosine vs. Control.

^b^SNP vs. Control.

^c^Values are n (%), except for MACE (pre-defined secondary outcome) for which %s are actuarial.

### Per-protocol analysis

In patients who actually received both doses of adenosine there was, when compared with controls, a significant increase in infarct size (%LVM, 12.0 vs. 8.3, *P* = 0.031) and LV volumes (LVEDVI [mL/m^2^], 91.4 ± 14.1 vs. 84.4 ± 14.6, *P* = 0.009) and EF (%) was reduced (42.5 ± 7.2 vs. 45.7 ± 8.0, *P* = 0.027). Major adverse cardiac events was increased in per-protocol adenosine-treated patients compared with control at 30 days (HR 5.91 [95% CIs 1.28–27.25], *P* = 0.036) and 6 months (HR 7.31 [95% CIs 1.62–33.0], *P* = 0.008). There was no increase in MACE in those who actually received SNP compared with controls (see Supplementary material online, *Table S9* and *Figure S1*).

### Sub-group analyses

In patients with anterior STEMI, those who received adenosine had significantly greater IS %LVM (16.2, 9.5–25.8 vs. 9.1, 1.8–13.4, *P* = 0.028) and extent of L-MVO %LVM (3.7, 0.5–6.1 vs. 0.3, 0.0–2.0, *P* = 0.008) compared with controls (see Supplementary material online, *Table S8*). Furthermore, the composite secondary clinical endpoint of death, MI, and heart failure was significantly greater in these patients (21.2 vs. 3.1%, *P* = 0.025) compared with controls.

## Discussion

Sub-optimal microvascular perfusion in STEMI confers additional morbidity and mortality despite TIMI 3 flow in the epicardial vessel.^25^ Adenosine and SNP are powerful vasodilators with pleiotropic effects, including anti-inflammatory, anti-platelet, and immune-modulatory actions, that have been shown to reduce IRI, particularly when delivered IC targeting the microvascular bed.^16^ However, our results show that neither adenosine nor SNP significantly reduced infarct size or MVO. Furthermore, IC adenosine appeared to be associated with detrimental cardiac effects and worse clinical outcome.

### Infarct size and markers of reperfusion injury

There was no significant difference in infarct size between the groups, although there was a trend to significant increase in infarct size in the adenosine group compared with controls when the results were adjusted for confounders. There was no reduction in MSI, early- or late-MVO measured by CMR with either drug. These results are consistent with the only other randomized trial using CMR, which found that high-dose IC adenosine did not increase myocardial salvage and did not reduce either infarct size or MVO.^9^ The per-protocol analysis showed that patients who received both doses of adenosine had significantly increased infarct size, LV volumes, and reduced EF on CMR compared with the control group, which suggests not only a lack of efficacy but potential cardiac toxicity and partial negation of any beneficial effects of reperfusion. This effect was particularly apparent in patients with anterior MI. Reasons for these adverse findings remain unclear but are considered below.

The proportion of patients achieving STR >70%, as an ECG surrogate marker of coronary vascular flow, was relatively high and similar across groups compared with previous studies.^8–11,17^ This may reflect our recruitment of only patients who presented within 6 h rather than within 12 h of symptom onset as in some previous studies.^7–9,17^ In REOPEN-AMI,^8^ STR >70% occurred in 71% of patients treated by adenosine, in 54% of patients treated by SNP, and in 51% of patients treated by saline (risk ratio: 1.39 [1.07–1.79]; *P* = 0.009, and risk ratio: 1.04 [0.78–1.40]; *P* = 0.75, adenosine or SNP vs. saline, respectively). Furthermore, in REOPEN-AMI, the enzymatic infarct size was 30% lower in the adenosine compared with the saline group but this is a greater effect size than would be expected by the attenuation of MVO alone, which makes interpretation difficult. However, in the absence of corroborative AAR or infarct size data from robust imaging such as CMR, this may imply that the adenosine group were destined to have smaller infarcts compared with control (saline), potentially over-estimating the benefit of adenosine in that study.

In our REFLO-STEMI trial, a positive impact of IC SNP on MVO assessed by ECG or CMR was not seen. This is consistent with the REOPEN-AMI study^8^ and an earlier double-blind, placebo-controlled RCT.^7^

The demonstration in our study, and in those of others,^3,26^ of failure of reduction in MVO or infarct size using adjunctive therapies, despite an extensive body of positive experimental data, raises the question of trial design or even whether IRI is a distinct entity clinically and whether it represents exclusively ischaemic injury.^26,27^

### Clinical outcome

There were significantly worse outcomes for the adenosine group compared with control, largely driven by increased early heart failure events. This is a novel finding. Whilst this may have occurred by chance, the hazard ratio is high and cannot be discounted. Our results, including definitive and significantly worse clinical outcome particularly in those with anterior STEMI receiving adenosine, contradicts the *post hoc* analysis of AMISTAD-II, which reported that a 3 h adenosine infusion decreased 1-month (5.2 vs. 9.2%, respectively, *P* = 0.014) and 6-month mortality (7.3 vs. 11.2%, *P* = 0.033) compared with placebo, and reduced the composite clinical endpoint of death or heart failure at 6 months (12.0 vs. 17.2%, *P* = 0.022).^28^ However, there were several methodological weaknesses with that study including: (i) adenosine was infused intravenously *after* the PCI, (ii) infarct size was measured in only 11% of patients and by technetium-99m sestamibi single-photon emission computed tomography, which may underestimate infarct size compared with CMR; and (iii) no measure of myocardial salvage was obtained. Furthermore, the adverse MACE signal seen with adenosine in our study is consistent whether assessed as intention-to-treat or per-protocol analysis, at 1 and 6 months follow-up and remains despite adjustment for potential confounding variables. This suggests that high-dose IC adenosine, delivered as we have indicated in this study, may lead to significantly worse clinical outcomes.

The finding of an adverse effect of high-dose adenosine remains difficult to explain. It could be that high-dose adenosine tends to activate other receptors, which lead to adverse events; adenosine has been speculated to be responsible for excess dyspnoea observed with ticagrelor in the PLATO study. However, any effect of ticagrelor on circulating adenosine levels is likely to be small compared with the relatively high doses of adenosine administered in our study. Furthermore, the distribution of ticagrelor and prasugrel across the treatment arms was comparable (see *Table 1*) and there were no significant differences in the primary endpoint or MVO for the patients receiving ticagrelor (see Supplementary material online, *Table S8*). Additionally, adenosine may mediate diuretic resistance; it acts on renal A_1_-AR on afferent arterioles to reduce glomerular flow and filtration, stimulate renin release, and enhance proximal tubular sodium reabsorption.^29^ It could be postulated that cross-activation of renal AR promoted fluid retention, increased LV volumes and advanced heart failure pathophysiology in our adenosine-treated cohort. Even though our finding is hypothesis generating, our data strongly suggest high-dose IC adenosine may lead to adverse events (possibly through cross-activation of other receptors) and probably should not be used to prevent MVO, in the circumstances of this trial.

### Limitations

The study was open-label, which may have influenced management of patients. However, the primary outcome was assessed on blinded CMR scans. The finding of an increased hazard signal with use of high-dose IC adenosine in our study must be interpreted with caution given our relatively small sample size. The power of the study may have been reduced due to the use of the FWHM technique, which has better observer variability but results in lower estimation of infarct size compared with previous studies using more conservative thresholds. Whilst we did not formally adjust for multiple hypothesis tests, which might not be considered ideal, this was a Phase II trial, and we did not want to exclude identifying potential efficacy of either pharmacological intervention. However, even if we had used a more stringent level of statistical significance, to account for testing both of our two active treatments relative to our control group, the statistical significance of our main results would remain unaltered. Similarly, although there was some evidence against the assumption of proportional hazards for the primary clinical outcome analysis (MACE by 6 months) this was not overwhelming, and our results were further supported by actuarial estimates of the absolute MACE event rate by 6 months.

It is possible that the dose of adenosine used in our study was insufficient to achieve sustained activation of A_2_-AR; ^30^ We utilized a slow-bolus dosing regimen and it could be that, owing to the short-half life of adenosine, concentrations of adenosine were insufficient in the microvascular bed to augment the benefits of reperfusion. A_1_-AR activation at lower adenosine doses may exacerbate MVO by promoting neutrophil chemotaxis.^30^ As such, delivering adenosine as a continuous infusion, to ensure more prolonged availability of adenosine in the microvasculature, may be superior to boli injections in antagonizing MVO-related processes. It should be noted that one might still consider low-dose (50–100 μg) adenosine, as is common in clinical practice, to reverse established slow-flow or no-reflow when it occurs.

## Conclusions

Intra-coronary adenosine and SNP did not reduce infarct size nor MVO during PPCI for STEMI. Furthermore, high-dose adenosine appeared to be associated with adverse clinical outcomes, increased infarct size, and reduced EF compared with control. These data suggest that neither agent is effective for sub-clinical no-reflow and should not be used routinely and prophylactically in the setting of PPCI to prevent reperfusion injury.

## Supplementary material

Supplementary material is available at *European Heart Journal* online.

## Authors’ contributions

S.N., K.A. performed statistical analysis; A.G., G.M., J.G., D.B., I.M., L.S., K.A., S.N. handled funding and supervision; S.N., J.K., G.M., J.G., D.B., V.K., M.B., A.G. acquired the data; A.G., G.M., J. G., D.B., I.M., L.S., S.N. conceived and designed the research; S.N., A.G. drafted the manuscript; G.M., J.G., D.B., V.K., J.K., I.M., M.B., R.W., A.A.J.A. made critical revision of the manuscript for key intellectual content.

## Funding

REFLO-STEMI was funded by the Medical Research Council (MRC) through the Efficacy and Mechanism Evaluation (EME) Board (project number 09/150/28) and managed by the NIHR on behalf of the MRC-NIHR partnership. Funding to pay the Open Access publication charges for this article was provided by The University of Leicester.

**Conflict of interest:** none declared.

## Supplementary Material

Supplementary Data
