# Supplementary material for: Strategies to attenuate micro-vascular obstruction during P-PCI: the randomized reperfusion facilitated by local adjunctive therapy in ST-elevation myocardial infarction trial
Source: Eur Heart J. 2016 May 4;37(24):1910–9. doi: 10.1093/eurheartj/ehw136 (PMC4917746; doi:10.1093/eurheartj/ehw136)
Supplement: Supplementary Data [file ehw136_supplementary_data.zip › ehw136supp.docx]

# SUPPLEMENTAL MATERIAL

Table 1: Eligibility Criteria

| **Inclusion Criteria** | **Exclusion Criteria** |
| --- | --- |
| - ≥ 18 years age. - Informed ASSENT (verbal consent) prior to angiography. - STEMI ≤ 6 hours of symptom onset, requiring PPCI. - Single-vessel coronary artery disease (non-culprit disease <70% stenosis at angiography). - TIMI flow 0/1 at angiography. - QTc <450ms. | - Contraindications to: PPCI, CMR, contrast agents or study medications - SBP ≤ 90mmHg - Cardiogenic Shock - Previous Q wave myocardial infarction - Culprit lesion not identified or located in a by-pass graft - Stent thrombosis. - Left main disease. - Known severe asthma. - Known stage 4 or 5 chronic kidney disease (eGFR<30ml/min/1.73m^2^). - Pregnancy. |

Table 2: TIMI Myocardial Perfusion Grade (TPMG)^1^

| **Grade of Myocardial Perfusion** | **Definition** |
| --- | --- |
| *TMPG 0* | Failure of dye to enter the microvasculature. |
| *TMPG 1* | Dye slowly enters but fails to exit the microvasculature. |
| *TMPG 2* | Delayed entry and exit of dye from the microvasculature. |
| *TMPG 3* | Normal entry and exit of dye from the microvasculature. |

Table 3: TIMI flow grade (TFG) Classification^2^

| **TFG** | **Definition** |
| --- | --- |
| *Grade 0* | No perfusion |
| *Grade 1* | Penetration without perfusion |
| *Grade 2* | Partial perfusion |
| *Grade 3* | Complete perfusion |

Table 4: TIMI thrombus score^3^

| **TIMI Thrombus score** | **Definition** |
| --- | --- |
| *0* | No characteristics of thrombus |
| *1* | Possible thrombus, as reduced contrast density, haziness, irregular lesion contour, or a smooth convex “meniscus” at the site of total occlusion suggestive but not diagnostic of thrombus |
| *2* | Definite thrombus, with greatest dimensions <1/2 vessel diameter |
| *3* | Definite thrombus, greatest linear dimension >1/2 but <2 vessel diameters |
| *4* | Definite thrombus, largest dimension >2 vessel diameters |
| *5* | Total occlusion |

Table 5: Definitions of adverse events

| **Adverse Event** | **Definition** |
| --- | --- |
| *Cardiogenic shock* | Systolic blood pressure <90 mmHg for at least 30 min (or the need for supportive measures to maintain a systolic blood pressure of >90 mmHg) in the presence of a heart rate of >60 beat/min in association with signs of end-organ hypoperfusion (cold extremities, low urinary output <30 mL/h and/or mental confusion). |
| *Myocardial infarction (MI)* | MI was defined differently in specific clinical situations in this trial. The European Society of Cardiology (ESC) and American College of Cardiology (ACC) criteria for acute, evolving or recent MI were applied^4^. |
| *Re-infarction* | Further chest pain during the index admission lasting >20 minutes accompanied by new electrocardiographic changes (new Q waves >0.04 seconds or ST-segment elevation >0.1 mV in two leads for >30 minutes), further enzyme rise or both. |
| *Recurrent MI* | A ≥20% rise in the value of the biomarker measured serially 6-12 hours apart, provided the absolute value was greater than the 99% percentile upper reference limit. For patients who died and for whom no cardiac markers were obtained, the presence of new ST segment elevation and new chest pain would meet criteria for MI. |
| *Contrast-induced nephropathy* | 25% increase in serum creatinine concentration from the baseline value, or absolute increase of at least 0.5 mg/dL (44.2 μmol/L), appearing within 48 hours of administration of contrast media, and maintained for 2–5 days^5-7^. |
| *Cerebrovascular Events* | Stroke was defined as a new focal neurological deficit of presumed vascular aetiology persisting > 24 hours combined with a neurological imaging study that did not indicate a different aetiology. Transient ischaemic attack (TIA) was any focal ischaemic neurological deficit of abrupt onset, which resolved completely within 24 hours. |
| *Severe Heart Failure* | *Early* heart failure: any new onset cardiogenic shock or heart failure occurring after randomization and during the index admission with radiographic evidence of pulmonary oedema requiring intravenous diuretic therapy.  *Late* heart failure: admission to hospital for treatment for documented New York Heart Association (NYHA) class III or IV heart failure. |
| *Major bleeding* | Defined according to the Thrombolysis in Myocardial Infarction (TIMI) criteria as fatal bleeding, any intracranial bleeding or clinically overt signs of haemorrhage associated with a drop in haemoglobin (Hb) of ≥ 50 g/L. |

Table 6: Study outcome measures

| **Type of outcome measure** | **Outcome measure** |
| --- | --- |
| ***CMR parameters*** | - CMR measured infarct size (% total LV mass) – **Primary outcome** - Incidence and extent of MVO (%LV mass) - Myocardial salvage index (MSI) - Intra-myocardial haemorrhage (IMH) - LV ejection fraction (LVEF) and volumes |
| ***Angiographic markers of MVO*** | - TIMI flow grade (TFG)^2^ - Corrected TIMI frame count (cTFC)^8, 9^ - TIMI Myocardial perfusion grade (TMPG) ^1, 10, 11^ - Computer-assisted myocardial blush quantification using the software ‘Quantitative Blush Evaluator’ (QuBE)^12^ - Incidence of angiographic slow/no-reflow after PPCI |
| ***ECG*** | - Degree of ST segment resolution on ECG^13, 14^ |
| ***Echocardiography*** | - LV function at baseline and 3 months |
| ***Sub-analyses*** | - Overall MACE and its components at 1 month: death, need for TLR, recurrent MI, severe heart failure and CVE |

CMR, cardiac magnetic resonance; CVE, cerebrovascular event; ECG, electrocardiogram; e-GFR, estimated glomerular filtration rate; LV, left ventricular; LVEF, MACE, major adverse cardiac events; MI, myocardial infarction; MVO, microvascular obstruction; PPCI, primary percutaneous coronary intervention; SBP, systolic blood pressure; TIMI, Thrombolysis in Myocardial Infarction; TLR, target lesion revascularisation

Table 7: Demography of the total trial population and the three CMR treatment groups

| **Characteristics** | **ADENOSINE** | | **SNP** | | **CONTROL** | | ***P* Value** | |
| --- | --- | --- | --- | --- | --- | --- | --- | --- |
|  | **All**  **(n=82)** | **With CMR**  **(n=63)** | **All**  **(n=79)** | **With CMR**  **(n=69)** | **All**  **(n=86)** | **With CMR**  **(n=65)** | **All** | **With CMR** |
|  |  |  |  |  |  |  |  |  |
| **Clinical** |  |  |  |  |  |  |  |  |
| Age, years | 57.9±12.8 | 56.4±12.3 | 60.5±13.0 | 59.3±12.5 | 59.5±11.2 | 60.0±10.8 | 0.406 | 0.192 |
| Male | 65/82 (79.3) | 52/63 (82.5) | 66/79 (83.5) | 59/69 (85.5) | 64/86 (74.4) | 50/65 (76.9) | 0.355 | 0.429 |
| Hypertension | 23/82 (28.0) | 14/63 (22.2) | 26/79 (32.9) | 23/69 (33.3) | 22/86 (25.6) | 16/65 (24.6) | 0.574 | 0.313 |
| Current smoking | 47/82 (57.3) | 39/63 (61.9) | 41/79 (51.9) | 37/69 (53.6) | 45/86 (52.3) | 31/65 (47.7) | 0.332 | 0.238 |
| Diabetes | 6/82 (7.3) | 3/63 (4.8) | 12/79 (15.2) | 10/69 (14.5) | 9/86 (10.5) | 4/65 (6.2) | 0.274 | 0.095 |
| Hypercholesterolaemia | 17/82 (20.7) | 15/63 (23.8) | 23/79 (29.1) | 17/69 (24.6) | 11/86 (12.8) | 6/65 (9.2) | **0.035** | **0.042** |
| Previous MI | 0/81 (0) | 0/63 (0) | 3/79 (3.8) | 3/69 (4.3) | 3/86 (3.5) | 2/65 (3.1) | 0.219 | 0.268 |
| Previous PCI | 0/82 (0) | 0/63 (0) | 3/79 (3.8) | 3/69 (4.3) | 2/86 (2.3) | 1/65 (1.5) | 0.225 | 0.197 |
| Killip class >1 | 3/82 (3.7) | 2/63 (3.2) | 4/79 (5.1) | 3/69 (4.3) | 4/86 (4.7) | 3/65 (4.6) | 0.905 | 0.908 |
| Total ischaemia time, min | 159 (124-221) | 155 (121-221) | 150 (122-201) | 150 (121-205) | 145 (105-196) | 141 (106-188) | 0.169 | 0.209 |
| BMI, kg/m^2^ | 27.5 (25.0-30.1) | 27.5 (25.1-32.1) | 26.1 (24.3-30.8) | 26.8 (24.7-31.0) | 27.3 (24.4-30.6) | 27.3 (25.1-30.7) | 0.659 | 0.833 |
| SBP, mm Hg | 137.5±25.1 | 136.2±25.4 | 133.0±23.4 | 132.9±23.3 | 135.1±23.3 | 136.2±23.5 | 0.505 | 0.663 |
| DBP, mm Hg | 86.6±19.2 | 86.0±18.8 | 81.8±16.4 | 82.3±16.4 | 80.5±17.0 | 80.6±17.8 | 0.067 | 0.215 |
| HR, beats/min | 74.0±17.3 | 72.8±18.8 | 71.9±14.8 | 72.3±14.9 | 71.2±14.6 | 71.3±14.7 | 0.487 | 0.878 |
| Cr clearance, ml/min/1.73 m^2^ | 98.1±28.1 | 101.6±26.4 | 93.4±29.1 | 93.8±30.0 | 92.7±25.6 | 93.9±26.1 | 0.401 | 0.190 |
|  |  |  |  |  |  |  |  |  |
| **Anti-platelet use** |  |  |  |  |  |  |  |  |
| Aspirin | 82/82 (100.0) | 63/63 (100.0) | 79/79 (100.0) | 69/69 (100.0) | 86/86 (100.0) | 65/65 (100.0) | 1.000 | 1.000 |
| Clopidogrel | 16/82 (19.5) | 11/63 (17.5) | 13/79 (16.5) | 11/69 (15.9) | 12/86 (14.0) | 9/65 (13.8) | 0.625 | 0.853 |
| Prasugrel | 38/82 (46.3) | 32/63 (50.8) | 33/79 (41.8) | 29/69 (42.0) | 40/86 (46.5) | 31/65 (47.7) | 0.790 | 0.590 |
| Ticagrelor | 28/82 (34.1) | 20/63 (31.7) | 33/79 (41.8) | 29/69 (42.0) | 34/86 (39.5) | 25/65 (38.5) | 0.591 | 0.468 |
|  |  |  |  |  |  |  |  |  |
| **Medication on admission** |  |  |  |  |  |  |  |  |
| Beta-blocker | 6/82 (7.3) | 4/63 (6.3) | 6/79 (7.6) | 6/69 (8.7) | 6/86 (7.0) | 3/65 (4.6) | 0.988 | 0.633 |
| ACE-inhibitor/A2RB | 14/82 (17.1) | 7/63 (11.1) | 17/79 (21.5) | 16/69 (23.2) | 13/86 (15.1) | 12/65 (18.5) | 0.549 | 0.190 |
| Statin | 17/82 (20.7) | 14/63 (22.2) | 25/79 (31.6) | 19/69 (27.5) | 13/86 (15.1) | 7/65 (10.8) | **0.036** | **0.049** |
|  |  |  |  |  |  |  |  |  |
| **Infarct-related artery** |  |  |  |  |  |  |  |  |
| LAD – proximal | 19/82 (23.2) | 11/63 (17.5) | 18/79 (22.8) | 16/69 (23.2) | 20/86 (23.3) | 15/65 (23.1) | 0.997 | 0.663 |
| LAD - other | 13/82 (15.9) | 12/63 (19.0) | 15/79 (19.0) | 12/69 (17.4) | 14/86 (16.3) | 12/65 (18.5) | 0.848 | 0.969 |
| LCX | 10/82 (12.2) | 6/63 (9.5) | 13/79 (16.5) | 13/69 (18.8) | 18/86 (20.9) | 12/65 (18.5) | 0.314 | 0.259 |
| RCA | 40/82 (48.8) | 34/63 (54.0) | 33/79 (41.8) | 28/69 (40.6) | 34/86 (39.5) | 26/65 (40.0) | 0.455 | 0.197 |
|  |  |  |  |  |  |  |  |  |
| **TIMI flow grade** |  |  |  |  |  |  |  |  |
| 0-1 | 80/81 (98.8) | 61/62 (98.4) | 72/79 (91.1) | 63/60 (91.3) | 83/86 (96.5) | 62/65 (95.4) | 0.057 | 0.180 |
| 2 | 1/81 (1.2) | 1/62 (1.6) | 5/79 (6.3) | 5/69 (7.2) | 1/86 (1.2) | 1/65 (1.5) | 0.078 | 0.124 |
| 3 | 0/81 (0.0) | 0/62 (0.0) | 2/79 (2.5) | 1/69 (1.4) | 2/86 (2.3) | 2/65 (3.1) | 0.367 | 0.368 |
|  |  |  |  |  |  |  |  |  |
| **Thrombus score** |  |  |  |  |  |  |  |  |
| 4-5 | 75/81 (92.6) | 57/62 (91.9) | 72/79 (91.1) | 64/69 (92.8) | 81/86 (94.2) | 60/65 (92.3) | 0.754 | 0.985 |
|  |  |  |  |  |  |  |  |  |
| Values are mean ± SD or n (%).  ACE-inhibitors/A2RB, angiotensin-converting enzyme inhibitors/angiotensin II receptor blockers; BMI, body mass index; CABG, coronary artery bypass graft; CAD, coronary artery disease; Cr clearance, creatinine clearance; DBP, diastolic blood pressure; HR, heart rate; LAD, left anterior descending coronary artery; LCX, left circumflex coronary artery; PCI, percutaneous coronary intervention; RCA, right coronary artery; SBP, systolic blood pressure; TA, thrombus aspiration; TIMI, Thrombolysis In Myocardial Infarction. | | | | | | | | |

Table 8: Main outcomes according to treatment group by stratifying criteria

| **Characteristic** | **Adenosine** | **SNP** | **Control** | **p Value*** | **p Value†** |
| --- | --- | --- | --- | --- | --- |
| **TTR <3h** | n=40 | n=43 | n=47 |  |  |
| Infarct Size, (%LVM) **$** | 9.7 (4.3-18.8) | 9.4 (3.2-15.8) | 6.4 (1.6-14.1) | 0.072 | 0.247 |
| L-MVO (%LVM) | 1.2 (0.0-4.6) | 0.4 (0.0-1.8) | 0.1 (0.0-2.9) | 0.146 | 0.466 |
| MSI, %, n | 61.1±25.3, n=19 | 70.8±17.2, n=25 | 69.1±22.9, n=28 | 0.266 | 0.759 |
| Composite of Death, MI and HF, n, % | 7/48 (14.6) | 3/50 (6.0) | 2/58 (3.4) | 0.075 | 0.661 |
|  |  |  |  |  |  |
| **TTR >3h** | n=23 | n=26 | n=17 |  |  |
| Infarct Size, (%LVM) **$** | 11.6 (7.0-15.0) | 11.4 (7.2-15.8) | 9.7 (4.3-13.2) | 0.525 | 0.462 |
| L-MVO (%LVM) | 0.5 (0.0-3.2) | 1.0 (0.1-3.4) | 0.7 (0.0-3.1) | 0.978 | 0.390 |
| MSI, %, n | 58.9±21.4, n=15 | 49.6±31.0, n=13 | 62.9±26.9, n=8 | 0.331 | 0.705 |
| Composite of Death, MI and HF, n, % | 4/33 (12.1) | 1/29 (3.4) | 0/27 (0.0) | 0.120 | 1.000 |
|  |  |  |  |  |  |
| **Anterior MI** | n=21 | n=27 | n=28 |  |  |
| Infarct Size, (%LVM) **$** | 16.2 (9.5-25.8) | 10.9 (5.0-18.7) | 9.1 (1.8-13.4) | **0.028** | 0.208 |
| L-MVO (%LVM) | 3.7 (0.5-6.1) | 0.6 (0.0-2.3) | 0.3 (0.0-2.0) | **0.008** | 0.393 |
| MSI, %, n | 57.7±20.3, n=12 | 71.0±17.1, n=20 | 70.9±21.5, n=18 | 0.103 | 0.993 |
| Composite of Death, MI and HF, n, % | 7/33 (21.2) | 1/35 (2.9) | 1/32 (3.1) | **0.025** | 1.000 |
|  |  |  |  |  |  |
| **Non-anterior MI** | n=42 | n=42 | n=37 |  |  |
| Infarct Size, (%LVM) **$** | 8.2 (4.0-13.5) | 9.4 (3.7-13.8) | 7.7 (2.2-14.3) | 0.473 | 0.435 |
| L-MVO (%LVM) | 0.5 (0.0-1.5) | 0.6 (0.0-3.2) | 0.3 (0.0-3.0) | 0.667 | 0.416 |
| MSI, %, n | 58.9±21.4, n=15 | 49.6±31.0, n=13 | 62.9±26.9, n=8 | 0.331 | 0.705 |
| Composite of Death, MI and HF, n, % | 4/49 (8.2) | 3/47 (6.4) | 1/51 (2.0) | 0.200 | 0.347 |
|  |  |  |  |  |  |
| **Pts receiving Ticagrelor** | n=20 | n=29 | n=25 |  |  |
| Infarct Size, (%LVM) **$** | 7.4 (2.7-14.5) | 9.7 (3.3-13.8) | 8.9 (3.2-13.3) | 0.929 | 0.944 |
| L-MVO (%LVM) | 0.3 (0.0-1.9) | 0.4 (0.0-1.9) | 0.1 (0.0-2.6) | 0.830 | 0.642 |
| MSI, %, n | 67.6±23.2, n=11 | 72.1±15.0, n=13 | 64.8±27.0, n=14 | 0.789 | 0.389 |
| Composite of Death, MI and HF, n, % | 5/28 (17.9) | 0/33 (0.0) | 1/34 (2.9) | 0.082 | 1.000 |
| Values are mean ± SD or median (interquartile range) unless otherwise stated.  *Adenosine versus Control. †SNP versus Control.  **$ Primary endpoint – comparison via independent t-test on log-transformed scale.**  HF, heart failure; L-MVO, late microvascular obstruction; LVM, LV mass; MI, myocardial infarction; MSI, myocardial salvage index; TTR, time to revascularisation | | | | | |

Table 9: MACE at 30 days and 6 months by ITT and Per Protocol

|  | | | **Hazard Ratio** | **95% CI** | **P-value** | **Log-Rank Test**  **P-value** |
| --- | --- | --- | --- | --- | --- | --- |
| ***1^st^ MACE within 30 days*** | | | | | | |
|  |  |  |  |  |  |  |
| **ITT** | **Unadjusted** | Adenosine *v* Control | 5.39 | (1.18 to 24.60) | **0.03** | **0.04** |
|  |  | SNP  *v* Control | 2.75 | (0.53 to 14.16) | 0.2 |  |
|  |  |  |  |  |  |  |
|  | **Adjusted*** | Adenosine *v* Control | 5.24 | (1.13 to 24.33) | **0.03** | **0.018** |
|  |  | SNP  *v* Control | 2.36 | (0.45 to 12.40) | 0.3 |  |
|  |  |  |  |  |  |  |
| **Per Protocol** | **Unadjusted** | Adenosine *v* Control | 5.91 | (1.28 to 27.25) | **0.02** | **0.036** |
|  |  | SNP  *v* Control | 3.12 | (0.57 to 17.04) | 0.2 |  |
|  |  |  |  |  |  |  |
|  | **Adjusted*** | Adenosine *v* Control | 5.76 | (1.21 to 27.35) | **0.03** | 0.1 |
|  |  | SNP  *v* Control | 2.94 | (0.52 to 16.60) | 0.2 |  |
| ***1^st^ MACE after 6 months median follow-up*** | | | | | | |
|  |  |  |  |  |  |  |
| **ITT** | **Unadjusted** | Adenosine *v* Control | 6.53 | (1.46 to 29.20) | **0.01** | **0.01** |
|  |  | SNP  *v* Control | 2.76 | (0.53 to 14.21) | 0.2 |  |
|  |  |  |  |  |  |  |
|  | **Adjusted*** | Adenosine *v* Control | 6.54 | (1.45 to 29.64) | **0.01** | **0.01** |
|  |  | SNP  *v* Control | 2.53 | (0.48 to 13.24) | 0.3 |  |
|  |  |  |  |  |  |  |
| **Per Protocol** | **Unadjusted** | Adenosine *v* Control | 7.31 | (1.62 to 33.00) | **0.01** | **0.008** |
|  |  | SNP  *v* Control | 3.14 | (0.57 to 17.13) | 0.2 |  |
|  |  |  |  |  |  |  |
|  | **Adjusted*** | Adenosine *v* Control | 7.32 | (1.59 to 33.59) | **0.01** | **0.05** |
|  |  | SNP  *v* Control | 3.20 | (0.57 to 18.01) | 0.2 |  |
| *** Adjusted for age, sex, diabetes, anterior MI, ischaemia time and Rentrop score.** | | | | | | |

**Figure 1: Kaplan-Meier graphs showing clinical outcome in the three treatment arms in patients treated per protocol (PP) at 30 days and 6 months**

# REFERENCES:

1. Gibson CM, Cannon CP, Murphy SA, Ryan KA, Mesley R, Marble SJ, McCabe CH, Van De Werf F and Braunwald E. Relationship of TIMI myocardial perfusion grade to mortality after administration of thrombolytic drugs. *Circulation*. 2000;101:125-30.

2. TIMI-Collaborators. The Thrombolysis in Myocardial Infarction (TIMI) trial. Phase I findings. TIMI Study Group. *The New England journal of medicine*. 1985;312:932-6.

3. Gibson CM, de Lemos JA, Murphy SA, Marble SJ, McCabe CH, Cannon CP, Antman EM, Braunwald E and Group TS. Combination therapy with abciximab reduces angiographically evident thrombus in acute myocardial infarction: a TIMI 14 substudy. *Circulation*. 2001;103:2550-4.

4. Thygesen K, Alpert JS, Jaffe AS, Simoons ML, Chaitman BR, White HD, Writing Group on the Joint ESCAAHAWHFTFftUDoMI, Thygesen K, Alpert JS, White HD, Jaffe AS, Katus HA, Apple FS, Lindahl B, Morrow DA, Chaitman BA, Clemmensen PM, Johanson P, Hod H, Underwood R, Bax JJ, Bonow RO, Pinto F, Gibbons RJ, Fox KA, Atar D, Newby LK, Galvani M, Hamm CW, Uretsky BF, Steg PG, Wijns W, Bassand JP, Menasche P, Ravkilde J, Ohman EM, Antman EM, Wallentin LC, Armstrong PW, Simoons ML, Januzzi JL, Nieminen MS, Gheorghiade M, Filippatos G, Luepker RV, Fortmann SP, Rosamond WD, Levy D, Wood D, Smith SC, Hu D, Lopez-Sendon JL, Robertson RM, Weaver D, Tendera M, Bove AA, Parkhomenko AN, Vasilieva EJ, Mendis S and Guidelines ESCCfP. Third universal definition of myocardial infarction. *Eur Heart J*. 2012;33:2551-67.

5. Reed MD and Bell D. Clinical pharmacology of bivalirudin. *Pharmacotherapy*. 2002;22:105S-111S.

6. Cortese B, Picchi A, Micheli A and Limbruno U. Intracoronary bivalirudin for no reflow reversal: a second chance to treat this disorder? *J Thromb Thrombolysis*. 2009;28:74-6.

7. Bertomeu-Gonzalez V, Bodi V, Sanchis J, Nunez J, Lopez-Lereu MP, Pena G, Losada A, Gomez C, Chorro FJ and Llacer A. [Limitations of myocardial blush grade in the evaluation of myocardial perfusion in patients with acute myocardial infarction and TIMI grade 3 flow]. *Rev Esp Cardiol*. 2006;59:575-81.

8. Gibson CM, Cannon CP, Daley WL, Dodge JT, Jr., Alexander B, Jr., Marble SJ, McCabe CH, Raymond L, Fortin T, Poole WK and Braunwald E. TIMI frame count: a quantitative method of assessing coronary artery flow. *Circulation*. 1996;93:879-88.

9. Kunadian V, Harrigan C, Zorkun C, Palmer AM, Ogando KJ, Biller LH, Lord EE, Williams SP, Lew ME, Ciaglo LN, Buros JL, Marble SJ, Gibson WJ and Gibson CM. Use of the TIMI frame count in the assessment of coronary artery blood flow and microvascular function over the past 15 years. *J Thromb Thrombolysis*. 2009;27:316-28.

10. Gibson CM, Cannon CP, Murphy SA, Marble SJ, Barron HV and Braunwald E. Relationship of the TIMI myocardial perfusion grades, flow grades, frame count, and percutaneous coronary intervention to long-term outcomes after thrombolytic administration in acute myocardial infarction. *Circulation*. 2002;105:1909-13.

11. van 't Hof AW, Liem A, Suryapranata H, Hoorntje JC, de Boer MJ and Zijlstra F. Angiographic assessment of myocardial reperfusion in patients treated with primary angioplasty for acute myocardial infarction: myocardial blush grade. Zwolle Myocardial Infarction Study Group. *Circulation*. 1998;97:2302-6.

12. Vogelzang M, Vlaar PJ, Svilaas T, Amo D, Nijsten MW and Zijlstra F. Computer-assisted myocardial blush quantification after percutaneous coronary angioplasty for acute myocardial infarction: a substudy from the TAPAS trial. *Eur Heart J*. 2009;30:594-9.

13. Schroder R, Dissmann R, Bruggemann T, Wegscheider K, Linderer T, Tebbe U and Neuhaus KL. Extent of early ST segment elevation resolution: a simple but strong predictor of outcome in patients with acute myocardial infarction. *J Am Coll Cardiol*. 1994;24:384-91.

14. Nijveldt R, Beek AM, Hirsch A, Stoel MG, Hofman MB, Umans VA, Algra PR, Twisk JW and van Rossum AC. Functional recovery after acute myocardial infarction: comparison between angiography, electrocardiography, and cardiovascular magnetic resonance measures of microvascular injury. *J Am Coll Cardiol*. 2008;52:181-9.
