# Supplementary figures and images for: Strategies to attenuate micro-vascular obstruction during P-PCI: the randomized reperfusion facilitated by local adjunctive therapy in ST-elevation myocardial infarction trial
Source: Eur Heart J. 2016 May 4;37(24):1910–9. doi: 10.1093/eurheartj/ehw136 (PMC4917746; doi:10.1093/eurheartj/ehw136)

PP: Time to 1st MACE (median follow-up 6 months)

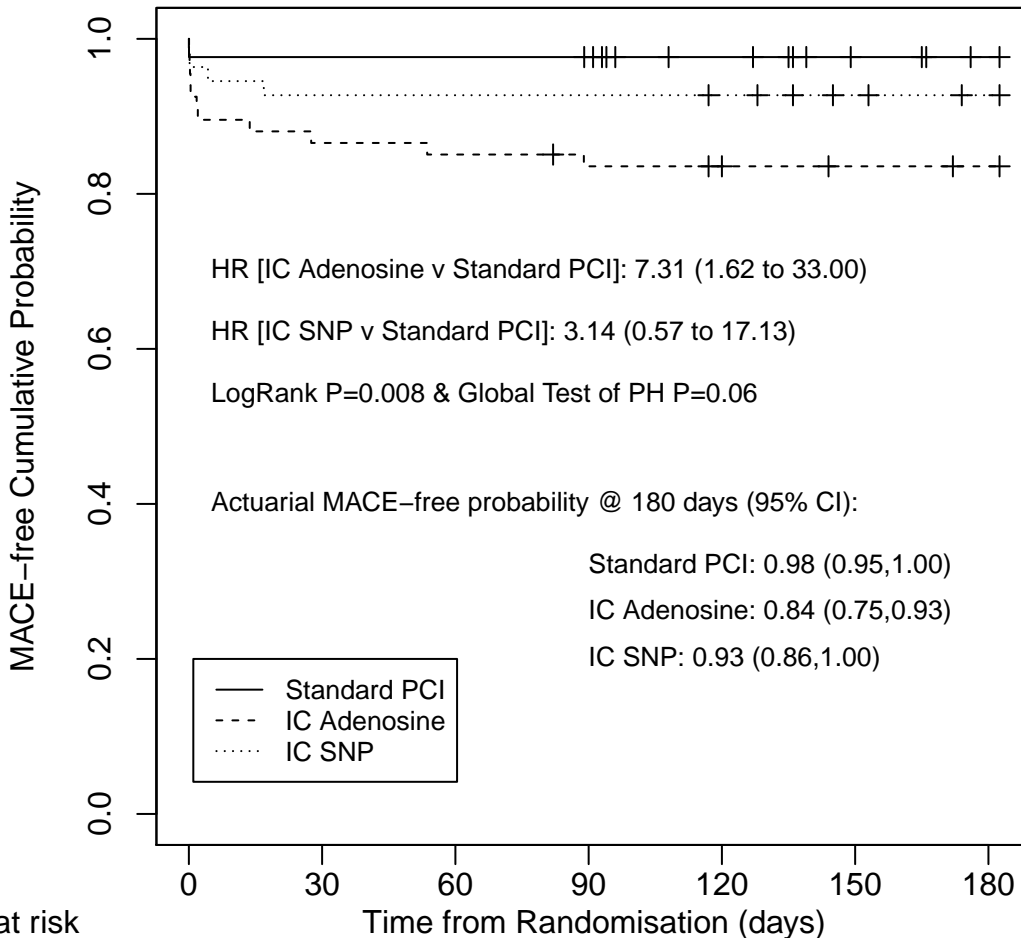

|              | 0  | 30 | 60 | 90 | 120 | 150 | 180 |
|--------------|----|----|----|----|-----|-----|-----|
| Standard PCI | 85 | 83 | 83 | 82 | 77  | 72  | 69  |
| IC Adenosine | 67 | 58 | 57 | 55 | 54  | 51  | 50  |
| IC SNP       | 55 | 51 | 51 | 51 | 50  | 47  | 44  |

Supplement: Supplementary Data [file ehw136_supplementary_data.zip › ehw136supp_fig1b.pdf]

PP: Time to 1st MACE within 30 days

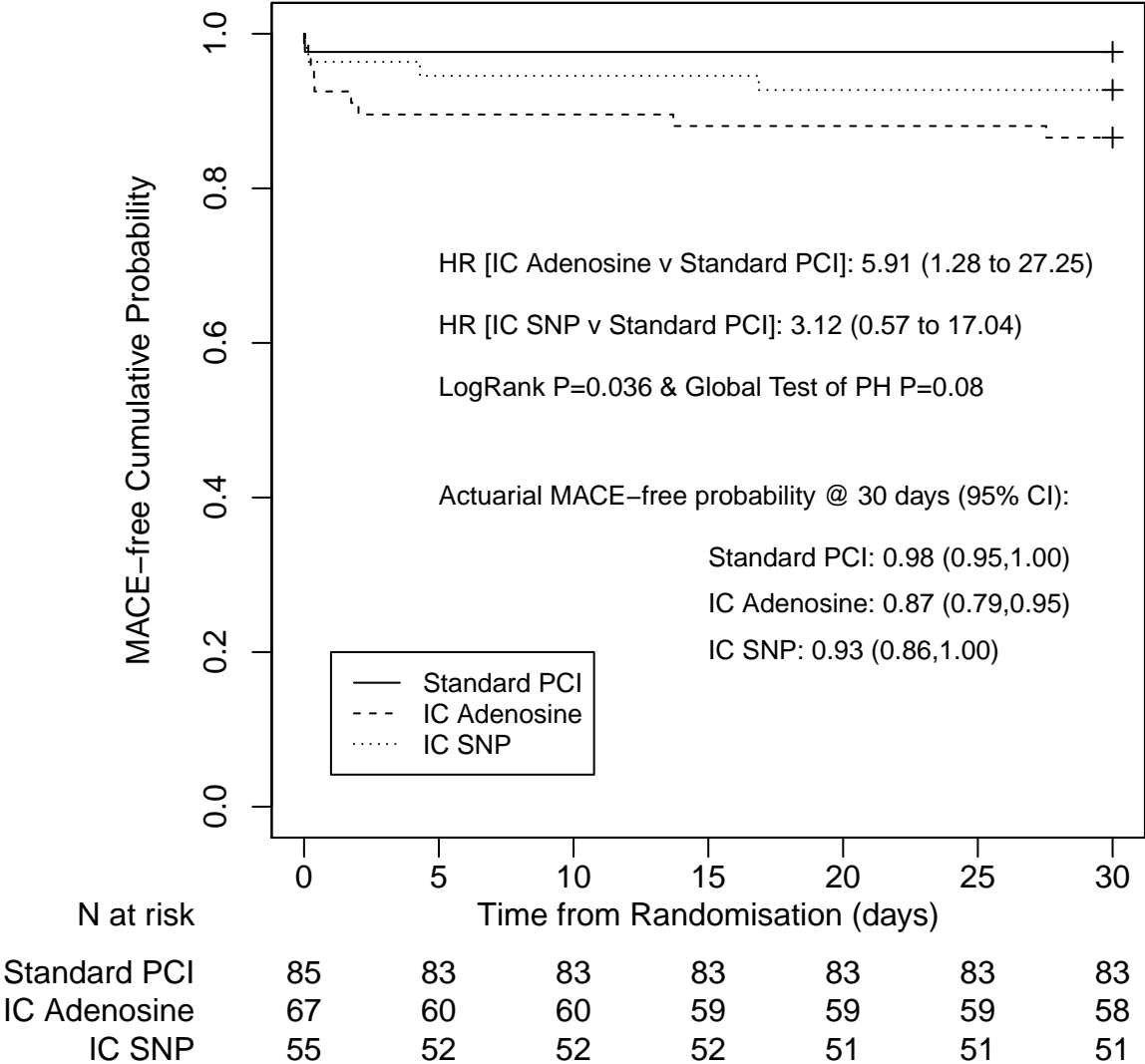

Supplement: Supplementary Data [file ehw136_supplementary_data.zip › ehw136supp_fig1a.pdf]
